# Leaching in Specific Facets of ZIF-67 and ZIF-L Zeolitic Imidazolate Frameworks During the CO_2_ Cycloaddition with Epichlorohydrin

**DOI:** 10.1021/acs.chemmater.2c03374

**Published:** 2023-01-03

**Authors:** Jose J. Delgado-Marín, Alejandra Rendón-Patiño, Vijay Kumar Velisoju, Gadde Sathish Kumar, Naydu Zambrano, Magnus Rueping, Jorge Gascón, Pedro Castaño, Javier Narciso, Enrique V. Ramos-Fernandez

**Affiliations:** † Instituto de Materiales and Departamento de Química Inorgánica, Facultad de Ciencias, Universidad de Alicante, Apdo. 99, Alicante 03080, Spain; ‡ KAUST Catalysis Center, Advanced Catalytic Materials, King Abdullah University of Science and Technology, Thuwal 23955, Saudi Arabia

## Abstract

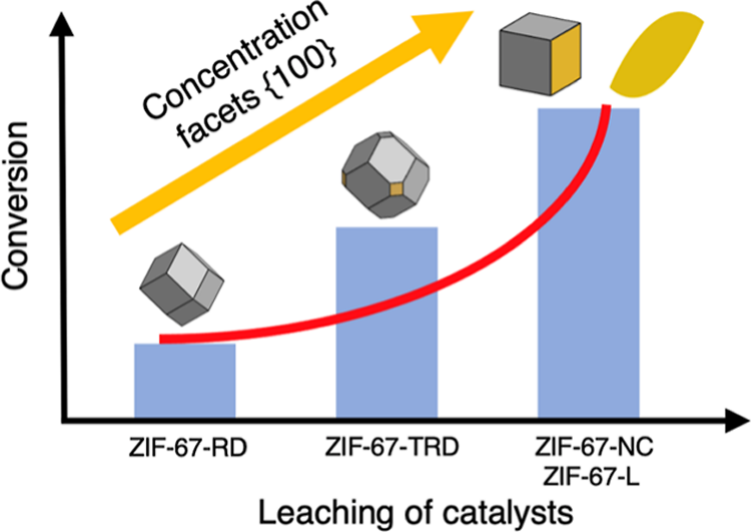

Zeolitic imidazolate frameworks (ZIFs) have been profusely used as catalysts for inserting CO_2_ into organic epoxides (i.e., epichlorohydrin) through cycloaddition. Here, we demonstrate that these materials suffer from irreversible degradation by leaching. To prove this, we performed the reactions and analyzed the final reaction mixtures by elemental analysis and the resulting materials by different microscopies. We found that the difference in catalytic activity between three ZIF-67 and one ZIF-L catalysts was related to the rate at which the materials degraded. Particularly, the {100} facet leaches faster than the others, regardless of the material used. The catalytic activity strongly depended on the amount of leached elements in the liquid phase since these species are extremely active. Our work points to the instability of these materials under relevant reaction conditions and the necessity of additional treatments to improve their stability.

## Introduction

In recent years, the scientific community has been working hard to find applications for metal–organic frameworks (MOFs).^1−3^ Among the most studied is their application as catalysts. MOFs are coordination polymers that, in principle, can mimic the behavior of homogeneous catalysts.^4^ One of their main limitations as catalysts is that the coordination bonds between the linker and the metal are relatively strong, and this prevents the reaction steps from taking place (dissociation, coordination, oxidative addition, reductive elimination, etc.).^5^ For this reason, the number of MOFs with potential application in catalysis is rather limited. In many cases, catalytically active centers have to be induced, inserted, or grafted to functionalize the structure.^6,7^

MOFs with intrinsic catalytic activity include those in which the metal centers are not fully coordinated, resulting in coordinatively unsaturated sites (CUS). This generates acidic Lewis centers. These materials have been used in epoxidation,^8,9^ Diels-Alder,^10^ or CO_2_ cycloaddition reactions with epoxides.^11^

**Scheme 1:**
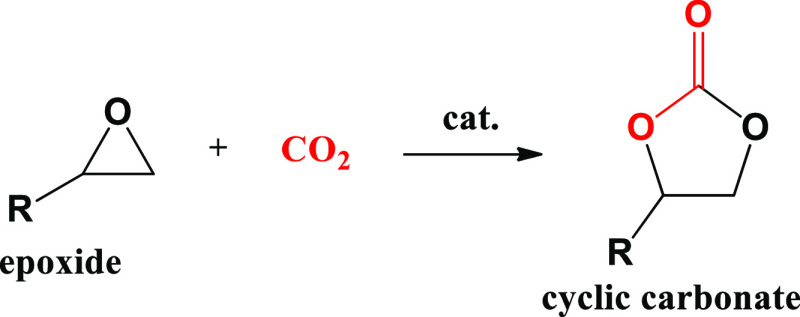
Synthesis of Cyclic Carbonates from an Epoxide

For CO_2_ cycloaddition (see Scheme 1), it is generally accepted that the Lewis acid sites are the active ones:^12^ the epoxide first binds to the Lewis acid via basic oxygen donation, followed by a nucleophilic attack by the co-catalyst to activate the epoxide. A standard co-catalyst is an ammonium halide, which forms haloalkoxies. This intermediate can further react with CO_2_ to form cyclic carbonate and regenerate the halide. In most cases, the nucleophilic attack is very fast, and the rate-limiting step is the coordination of the epoxide with the Lewis acid site.^13−16^

There are several MOFs that do not have Lewis acid sites in either the linker or the metal cluster, and yet these are remarkably active in CO_2_ cycloaddition reactions, even without a co-catalyst. In these cases, the reaction must be catalyzed by defects in the crystal structure. Farha et al.^17^ have termed them as “MOFs with opportunistic active sites”. The fact that the reaction proceeds without a co-catalyst suggests a bi-functional character of these materials with Lewis acidic and basic sites.^17^

The ZIF-8 and ZIF-67 structures were the first to be discovered as having the mentioned opportunistic active sites. In fact, both materials are very active in cycloadditions.^1−3,18^ ZIF-8 and ZIF-67 are constructed with 2-methylimidazole (2-MIM) and Zn^2+^ or Co^2+^, respectively. Both Zn and Co are tetrahedrally coordinated and fully saturated with the nitrogen moieties of the linker. There are no CUSs or catalytically active organic functional groups in the linker. In addition, the pore opening of the structure is around 0.3 nm, which makes it difficult for large molecules to diffuse.^19,20^ Considering all these facts, someone would anticipate that ZIF-67 and ZIF-8 materials should not be active for cycloadditions, yet they are proved to be active even in the absence of a co-catalyst and without a solvent. This contradiction has triggered a number of fundamental research questions that should be answered in order to understand how these catalysts work. Most of the published works using these materials as catalysts attribute the origin of their catalytic activity to defects on the outer surface of the crystal.

In these MOFs, each crystal shape has different exposed crystallographic planes, and each crystallographic plane has a distinct chemical composition. In ZIF-67, the {100} and {211} planes contain several Zn-2-MIM linkages, whereas the {110} and {111} planes do not contain any of these linkages. In this context, the facets {100} and {211} planes are expected to enhance the catalytic reaction, thanks to a higher concentration of surface defects.^21,22^ Given that the catalysis of these materials might occur on the outer surface of the crystal, it is reasonable to hypothesize that by controlling the composition and structure of the crystal surface, we can tune the catalytic activity of these materials.

The stability of these materials has been the subject of debate for a long time.^23,24^ The {100} facet of these MOFs is more unstable in corrosive environments than the other facets.^25^ It is therefore important to analyze these materials after they are used as catalysts. In many cases, the degradation is slow, and it cannot be detected by analyzing the material after the reaction using conventional techniques such as X-ray diffraction (XRD) or N_2_ adsorption. Thus, even if a significant portion of the catalyst degrades, no degradation is observed.^26−28^

In this work, we have prepared four crystals of ZIF-67 and ZIF-L with different orientations to unravel the effect of the exposed facets on the catalytic activity during CO_2_ cycloaddition. Our objective is to reveal the true nature of the opportunistic active sites of these materials. To our surprise, we found that these materials degraded very quickly and severely under reaction conditions. We also identified that the degradation of the materials used in this work was dependent on the exposed facet. The {100} side degrades faster than the other sides of the crystal. We extended this work to another ZIF-L structure, which also has the {100} side preferentially exposed. Similar results were found.^21,25,29,30^

## Result and Discussion

We used different protocols to prepare three types of ZIF-67: nanocubes (ZIF-67-NC), dodecahedral rhombic (ZIF-67-RD), and truncated dodecahedral rhombic (ZIF-67-TRD). All three samples show the same diffraction pattern, as depicted in Figure S1. The pattern corresponds to the sodalite structure of this type of zeolitic imidazolate framework (ZIF) and matches very well with the literature.^3,31−35^

ZIF-L was also prepared from the same linker and metal. ZIF-L exhibits a two-dimensional structure with periodic laminar alignment and free linkers remaining between the lamellae (to stabilize the structure).^33,34,36^ This structure was used because of its laminar character and the fact that crystals terminate at the linker. XRD analysis (Figure S1) demonstrates the successful synthesis of this structure.^37^

The morphology of the particles was checked by scanning electron microscopy (SEM, Figure S2). The observed morphology is different among samples, varying from very well-defined nanocubes to dodecahedral rhomboids for ZIF-L, indicating different facet orientations.

**Figure 1:**
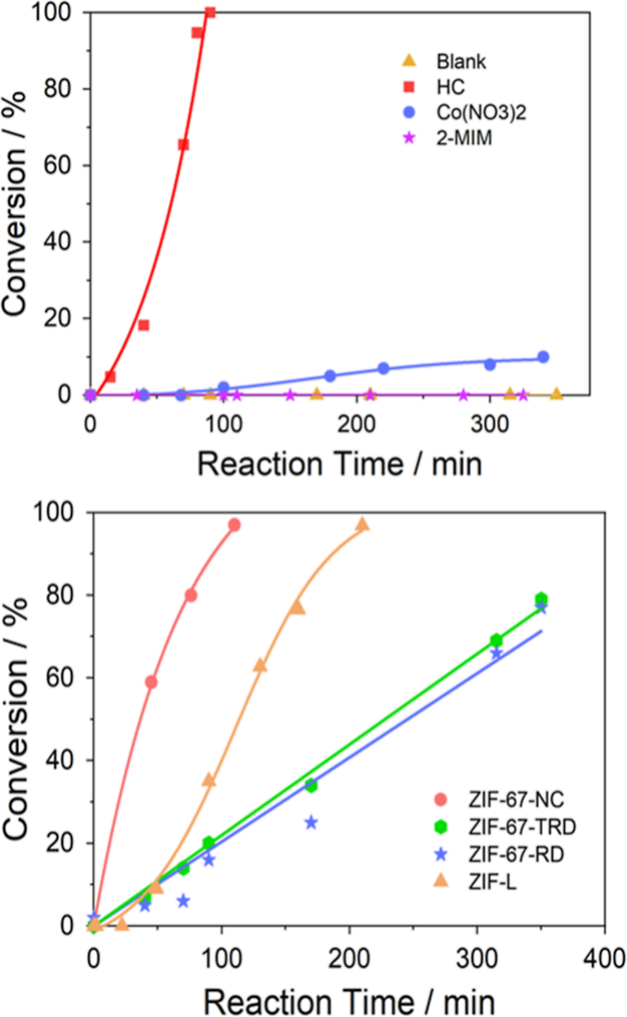
Two graphs show how the conversion of epichlorohydrin varies with time, when the reaction is done at 120 °C, 15 bar CO_2_, and a stirring speed of 1000 rpm. Top graph shows the control experiments. The orange triangles are when the reaction is done without the catalyst. The violet stars are when the reaction is catalyzed with 0.17 g of 2-MIM. The blue circles are when 0.63 g of cobalt nitrate is used as the catalyst. Red squares represent the reaction catalyzed by a mixture of 2-MIM (0.17 g) and cobalt nitrate (0.63 g). Button graph shows the catalytic behavior of the different samples prepared.

Before performing the catalytic tests, a series of control experiments were carried out (Figure 1). These include experiments (i) without catalysts, (ii) only in the presence of the linker (2-MIM), (iii) only with Co^2+^, or (iv) with a mixture of Co^2+^ and 2-MIM [named as (HC)]. When Co^2+^ is added, the reaction proceeds slowly, which shows that Co^2+^ alone is not a good catalyst for this reaction, 10% conversion was found after 350 min. 2-MIM alone does not catalyze the reaction, and no conversion was detected after the 350 min reaction. When a mixture of Co^2+^ and 2-MIM in stoichiometric amounts is used as a catalyst, the epoxide is consumed in only 100 min. Moreover, in the first 90 min, only organic carbonate is produced (100% selectivity), and only at high conversions are by-products (polycarbonates) observed in the 1H MNR spectra. When calculating the turnover number (TON) (mole of epoxide consumed per mole of Co^2+^) we found values of 331 and a turnover frequency of 0.058 s^–1^, these values are in the range of homogeneous catalysts based on Salen compounds.^38^ Furthermore, after opening the reactor, we saw that there was a dark brown precipitate. This precipitate was analyzed by SEM and was found to consist of a mixture of spheres and other small crystal-like particles (Figure S3).

These results put us on alert, as small amounts of ZIF components leached into the reaction mixture would give misleading results. When we test four prepared samples, we can see that the ZIF-67-NC performs as well as HC. ZIF-L catalyzes the reaction; however, there is an induction time before the conversion starts to grow rapidly. The ZIF-67-RD and ZIF-67-TRD samples show a linear reaction over time, which indicates that the reaction is zero order and independent of the concertation of reagents. These autocatalytic and zero-order kinetics are rarely found in this type of MOFs. The induction time of an autocatalytic reaction is usually related to a change in the structure (toward a more active one) or to the formation of an intermediate that co-reacts faster with the reagent. In the case of the ZIF-67-RD and ZIF-67-TRD catalysts, the zero-order indicates either that we have diffusional problems (gas liquid mass transport or gas solid-mass transport) or any other artifact is controlling as this kinetics has not been described in the literature for this reaction and for relatively similar catalysts.

In reactions carried out in batch reactors, it is difficult to identify whether the catalyst is deactivated or changed during the experiment. For this reason, the used catalysts (after reaction, thorough washing with ethanol, and drying at 80 °C overnight) were characterized by infrared spectroscopy and X-ray diffraction.

**Figure 2:**
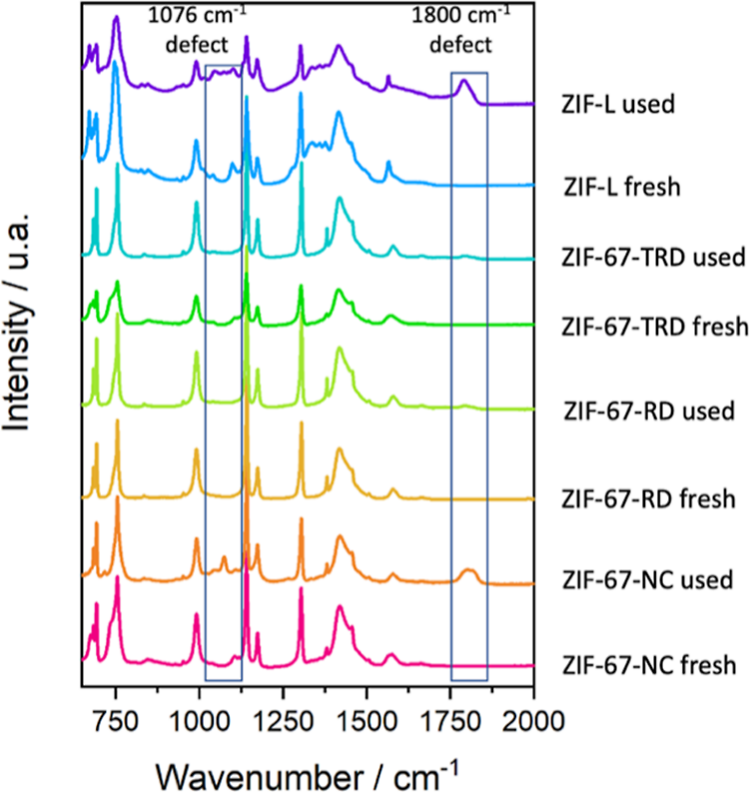
Infrared spectra of all the samples before and after being used.

**Figure 3:**
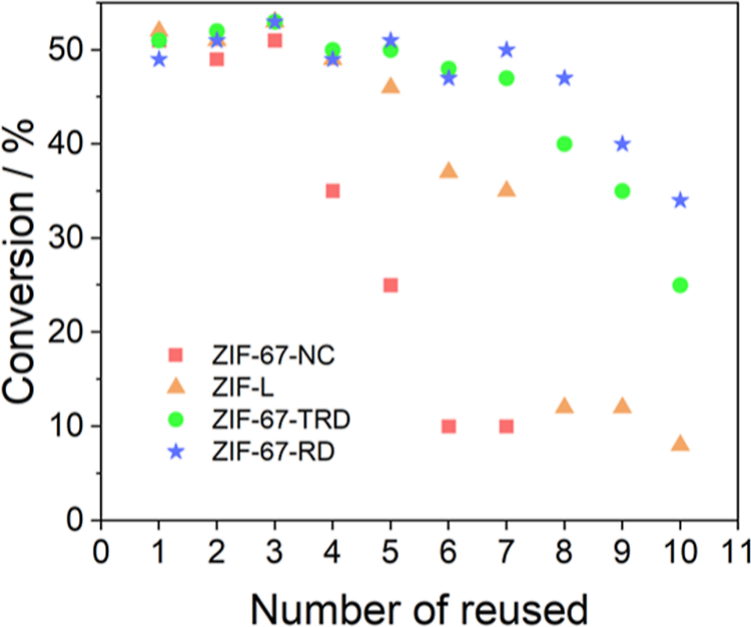
Reusability tests.

The diffraction patterns of the samples before and after use do not show any significant differences (Figure S1). However, a closer analysis of the infrared spectra before and after use (Figure 2) reveals small changes. All samples used in reaction (including ZIF-L) show two more peaks than their original fresh counterparts. One peak is centered at 1076 cm^–1^ and the other at 1800 cm^–1^. The vibrational mode at 1076 cm^–1^ peak is associated with asymmetric in-plane ring stretching of the uncoordinated linker 38 and the resonance between the N–H...N bending out of plane and the N–H stretching vibrations.^39^ Both peaks are also present in the infrared spectrum of 2-methylimidazole.^40^ These infrared peaks show that defects are generated in the material due to, but not exclusively, Co^2+^ leaching and that uncoordinated bonds remain in the ZIFs. This is the first evidence we have found that the materials may be degrading. Another difference we found between different samples is that for the two most active samples, ZIF-L and ZIF-67-NC, the peaks at 1800 and 1076 cm^–1^ are more intense than in the samples ZIF-67-RD and ZIF-67-TRD. This indicates that the most active samples are also the ones that degrade faster. To further check the stability of these materials, several additional reuses were carried out for all catalysts (Figure 3). The reaction was stopped, and the catalyst was filtered, washed with ethanol, dried, and used again. The reaction time at which the reaction was stopped was the time at which 50% conversion was achieved in the first use. This process was repeated up to 10 times. In this way, we could see how the conversion decays over the number of uses. As we can see, the ZIF-67-NC catalyst shows a significant drop in conversion in the fourth cycle and total activity loss after six cycles. Similar behavior occurs for all the other catalysts, and as the initial activity of the catalyst lowers, so does its deactivation rate: ZIF-67-TRD and ZIF-67-RD catalysts have lower activity (Figure 1) and slower deactivation (Figure 3).

**Figure 4:**
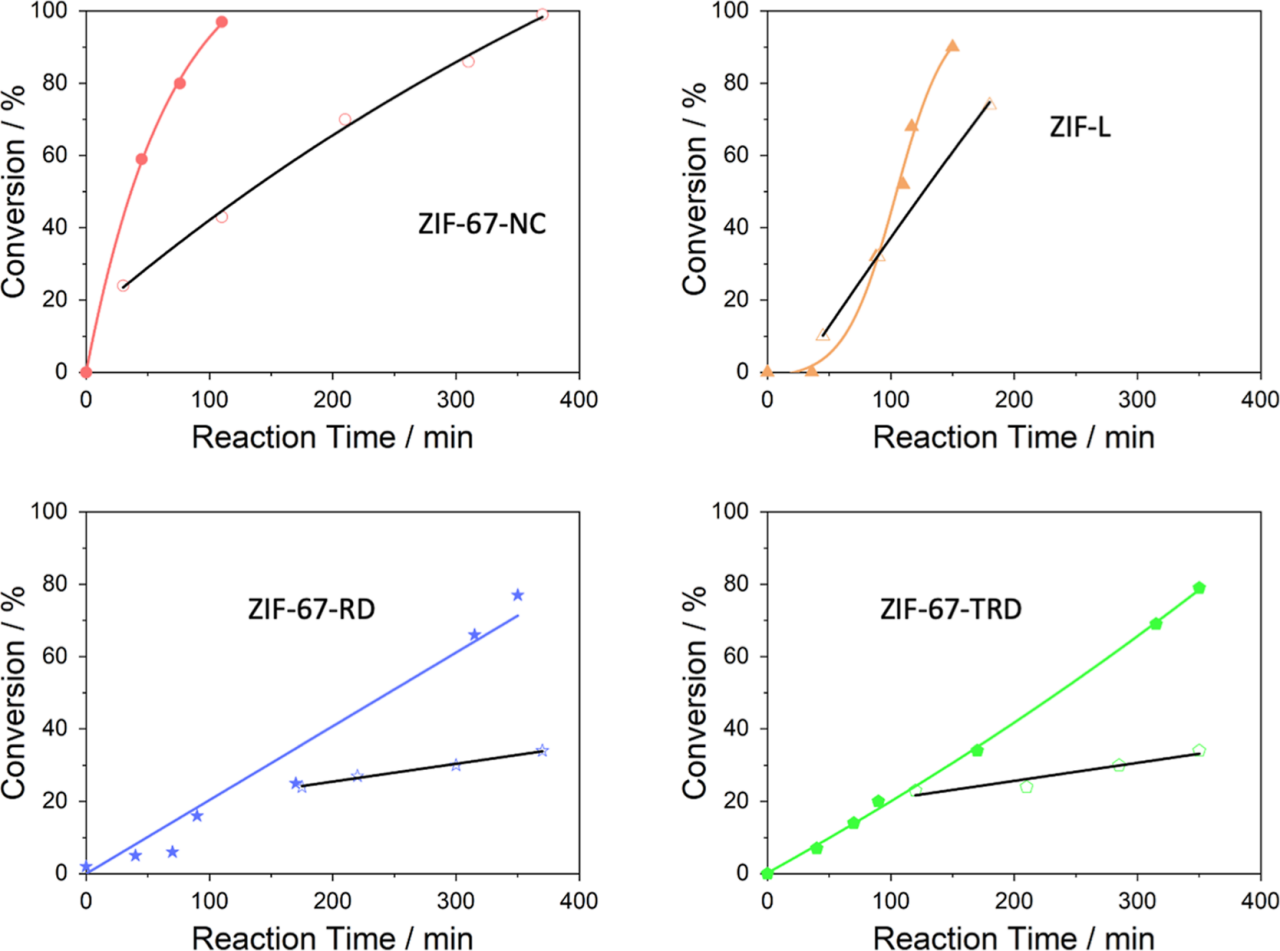
These four graphs show the catalytic filtering experiments. The filled symbols show an experiment at 120 °C and 15 bar CO_2_, where the catalyst is always present in the reaction. The empty symbols start at the point in the reaction where the catalyst has been removed from the reaction mixture and remains in the reaction conditions. In all cases, it is seen that after the removal of the catalyst, the reaction proceeds without the catalyst.

Our results clearly show that all the catalysts degrade over time and cycle. The most typical deactivation causes are fouling, site degradation (leaching, sintering... etc.), and poisoning. Fouling, sintering, and poisoning can be discarded given the mild reaction conditions and the absence of activity modifiers (use of pure reagents). This leaves leaching and site degradation as the most probable causes of deactivation. To prove that, we have carried out hot filtration experiments. In these experiments, when 50% conversion is reached, the catalyst is removed from the reaction mixture and the reaction is continued without the solid. If the reaction proceeds without a catalyst, it means that catalytically active species have leached out of the catalyst. Figure 4 shows these results, and as can be seen, the liquid samples extracted from the products of ZIF-L and ZIF-67-NC catalysts are very active, which proves that they leach, although at lower rates than when the catalyst is present due to the fact that the number of leached active sites is lower than the ones present in the catalyst. This indicates that a continuous supply of leached species is needed for the reaction to be kinetically maintained. When we look at the hot filtration experiments for the ZIF-67-TRD and ZIF-67-RD samples, we see that the conversion rate only increases by 7 and 5%, respectively, after the catalyst is removed. This indicates that the leaching in these samples is lower than in the two most active samples.

**Figure 5:**
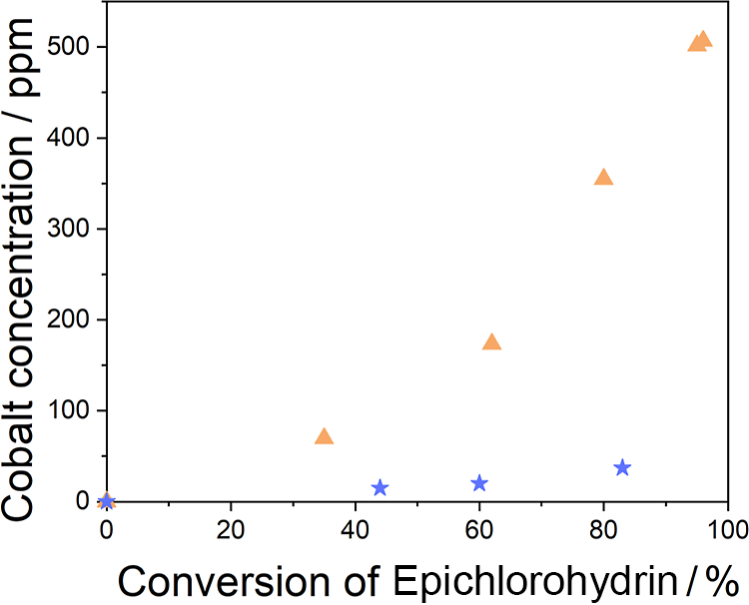
Concentration of cobalt in the reaction mixture versus conversion of epichlorohydrin. Yellow triangles appear when the ZIF-L sample is used as the catalyst. Blue triangles appear when ZIF-67-RD is used.

To check that leaching was occurring, we analyzed the mixture at different reaction times by ICP–MS and confirmed that the concentration of coin solution increased with conversion in a quasi-linear fashion (Figure 5). For this, we focused on two samples ZIF-L and ZIF-67-RD. These two were chosen because ZIF-L degrades after a few cycles, and in the experiment, the reaction proceeds when the catalyst has been removed. In contrast, ZIF-67-RD needs 10 cycles to lose catalyst performance, and there is no large increase in conversion after the catalyst is removed from the reaction. As can be seen in the ZIF-L sample, the amount of Co^2+^ increases in the reaction mixture as the reaction proceeds. Furthermore, it can be seen that in the first moments of the reaction there is no Co^2+^ leachate. The Co^2+^ concentration in the reaction mixture has the same trend as the conversion. This explains the significant catalytic activity of this material as well as the induction time. The ZIF-L catalyst needs some time to start degrading. This confirms that leaching occurs and that catalytic activity results from the leached species rather than from the heterogeneous catalyst.

When using the ZIF-67-RD catalyst, during the first moments of the reaction, no Co^2+^ species are detected in the liquid medium (Figure 5), but after a while, the Co^2+^ concentration starts to rise (the concentration of cobalt is 1 order of magnitude lower than the one found in ZIF-L). This explains why conversion grows over time in a linear fashion since it is actually directly related to the rate at which species are leached from the catalyst into the reaction mixture.

These results contradict a number of literature studies.^1,2,17,18,37,38,40−45^ However, when going in detail through these works, we noticed that, in most cases, the number of reuses was limited to 4. On the other hand, most of the studies in the literature do not specify the type of crystal used, RD or TRD. However, from the reported synthesis methods, we can infer that most of the studies probably used RD, as this is the thermodynamic product. This makes it even more difficult to detect the leaching of the crystal species. The RD crystals do not have the {100} side exposed, and therefore, their degradation is slower.

The first authors to find Co in the reaction mixture were Verpoort et al.^1,2^ who found 0.2% Co in solution, although they deemed this concentration irrelevant and did not provide sufficient data as to how they calculated it (i.e., we have seen a large difference when performing hot filtration experiments that do not allow for precipitation of Co species in solution (see control experiments above). To the best of our knowledge, this is the only article reporting a study on leached species. This is very surprising, as hot filtration experiments are essential when performing liquid phase reactions.

Doonan et al.^21^ recently conducted a study on the use of ZIF-8 as a catalyst in transesterification and Knoevenagel condensation. ZIF-8 has been considered a good transesterification catalyst, and its catalytic properties have always been ascribed to surface defects. These authors found that during the reaction, the crystal size of these catalysts decreased with time, and by AA-AES, they found that the catalytic activity was directly related to the rate at which Zn^2+^ leached. The same was found for the Knoevenagel reaction. This study already points out the precautions we have to take when using this type of ZIFs as liquid phase catalysts.

**Figure 6:**
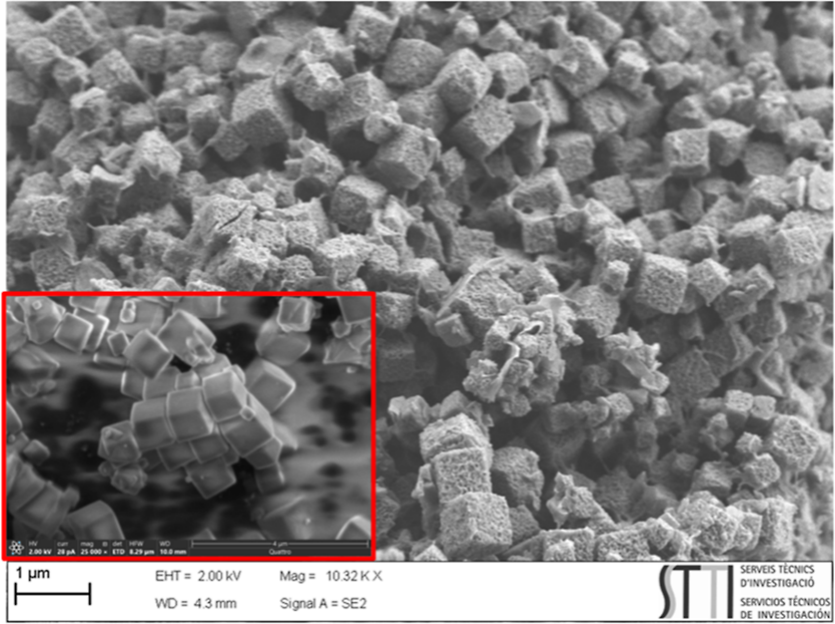
SEM image of the ZIF-67-NC after being used once as a catalyst. The inset of the figure is the image of the ZIF-67-NC as prepared (before any reaction run).

To understand why some materials degraded faster than others, consequently increasing the reaction rate, SEM images were taken for all samples after their first use as catalysts in the reaction. For the samples ZIF-L and ZIF-67-RD, TEM was also carried out. As can be observed in Figure 6, the crystals of sample ZIF-67-NC are still cube-shaped, but it appears that these cubes are filled with cavities and holes, forming a cube-shaped foam. This is far from the morphology of the crystals before they were used (see the inset of Figure 6).

**Figure 7:**
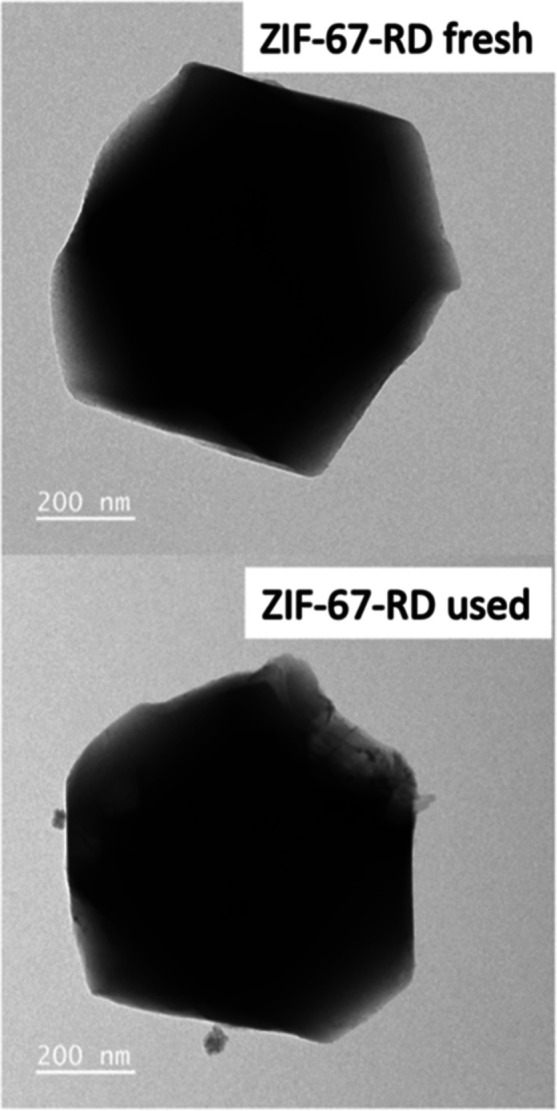
TEM images of the ZIF-67-RD sample before and after being used.

When we look at the ZIF-67-RD sample (Figure 7), we see that most of the crystals seem to be undamaged, but if we analyze the images in detail, we see that in the vertices of some crystals, there is a small hole in Figure S4. This vertex corresponds to the {100}, which is the same as what the crystals of ZIF-67-NC have exposed.^25^ This indicates that this is the most unstable facet and where the degradation of the crystals begins. Hence, the ZIF-67-RD sample shows less leaching, as it has less facet surface {100} exposed, while ZIF-67-NC has only {100} on its external surface. To corroborate that the ZIF-67-RD sample degraded preferentially through the {100} facet, TEM images were taken (Figure 6). As can be seen, the ZIF-67-RD crystals have the expected shape before being used in reaction, and when used in reaction, it can be seen that through the {100} facet, the sample starts to degrade. This degradation does not occur in all crystals at the first use. However, it is enough to accelerate the reaction.

**Figure 8:**
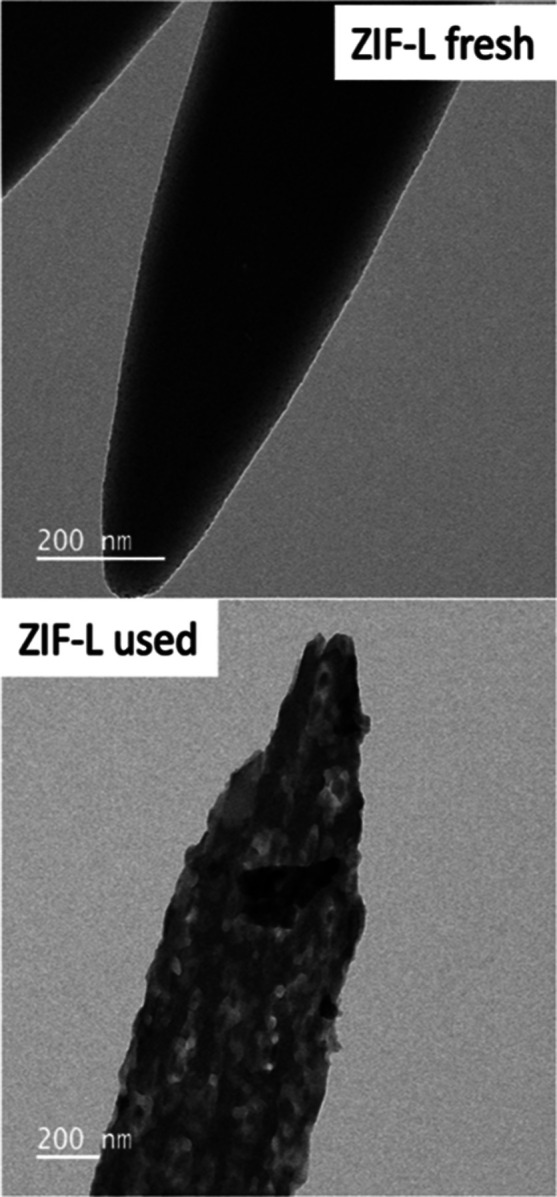
Top graph shows the ZIF-L fresh bottom graph.

If we look at the ZIF-L sample, we can see how the surface goes from being totally smooth to rough (Figure 8), also indicating that the sample degrades and leaches species into the reaction mixture. As we have indicated before, the surface of these crystals is full of linkers partially coordinated to the metal. When these linkers are washed off the surface, the {100} facet of the ZIF-L, which is identical to that of the ZIF-67, is exposed, and the damage to the crystal becomes more evident. This explains the induction time, which is due to the washing of 2-MIM partially co-ordinated to the metal. TEM images corroborate this fact (see Figure 7)

## Conclusions

ZIF materials are not the actual catalysts when applied in CO_2_ cycloaddition. Instead, we found that the leaching of some constituents of the MOF results in the formation of species in solution that are much more active than the MOF itself. In addition, the exposed facets on the MOF crystals play a large role, with the {100} facet degrading much faster.

Our results further stress the importance of in-depth analysis prior to claiming catalytic activity: to avoid “apparent” stabilities, abundant reuse and hot-filtration experiments are required, along with a detailed characterization of the used materials and reaction media.

## Experimental Section

### Synthesis

The ZIF-67 nanocubes (ZIF-67-NC) were prepared according to the ref (46). Thus, 580 mg of Co(NO_3_)_2_·6H_2_O was dissolved in 20 mL of deionized water containing 10 mg of cetrimonium bromide. Then, this solution was rapidly injected into 140 mL of aqueous solution containing 9.08 g of 2-methylimidazole and stirred at room temperature for 20 min. The product was collected by centrifugation and washed with ethanol six times.

The synthesis of truncated (ZIF-67-TRD) and non-truncated rhombic dodecahedral ZIF-67 crystals (ZIF-67-RD) was done following the recipe published elsewhere.^25^ A solution of 6 g of Co(OAc)_2_·4H_2_O in 50 mL of DI water was added into a solution of 22.4 g of 2-MIM in 50 mL of DI water, and the resulting mixture was homogenized by stirring it for a few seconds. Then, the mixture was left at room temperature for 10 min to form truncated rhombic dodecahedral ZIF-67 crystals. Non-truncated rhombic dodecahedral ZIF-67 crystals were prepared using the same procedure as the truncated ones, except that the mixture was left for 5 h at room temperature.

The ZIF-L sample was prepared by mixing two solutions. One contained 2.62 g of 2-MIM dissolved in 80 mL of water, and the other 0.78 g of Co(NO_3_)_2_·6H_2_O was dissolved in 80 mL of water. After mixing with a magnetic stir bar for 5 min, the solution was left static for 3 h.

### Characterization

X-ray diffraction analysis was used to identify the crystallographic phases of the samples. PXRD was recorded on a Bruker diffractometer with a goniometer that had an X-ray tube (Kα, λ = 1.54 Å) fitted with a Cu cathode and a detector PIXcel 3D. The range of the spectra was registered between 0.5 and 70° with a step size of 0.01° and a step time of 20 s.

To study the leaching of Fe during the reaction, inductively coupled plasma mass spectrometry was used. The analysis was obtained on ICP–MS 8900 Agilent equipment. For the preparation of the samples, 100 μL of the final reaction product mixture was diluted in 5 mL of distilled water and shaken to mix homogeneously. The ICP was calibrated with standard solutions.

The Fourier-transform infrared (FT-IR) spectra were obtained using a Jasco FT-IR 4700 (detector DLaTGS) in ATR mode with a diamond crystal for the measurement of solid samples. The spectral range was from 500 to 4000 cm^–1^ monitored with a resolution of 1 cm^–1^.

Scanning electron microscopy (SEM) images of the samples were acquired with a FEI Teneo VS microscope (FEI Company, Hillsboro, OR, USA). The electron beam was accelerated at 2 kV and 10 nA, and the images were acquired at around 3 mm working distance. Transmission electron microscopy (TEM) micrographs were obtained with a Titan ST microscope (FEI Company, Hillsboro, OR, USA) operating at 300 kV. Dry sample preparation was used for all of the samples onto copper grids coated with a carbon film (200 mesh).

For the catalytic tests, the samples were previously activated by heating at 150 °C overnight to remove the guest molecules such as water or ethanol. To carry out the reaction, epichlorohydrin (33 g), mesitylene as the NMR internal standard (0.4 g), and the activated catalyst (0.23 g) were introduced in a steel vessel with a magnetic stirrer. After that, the reactor was closed and purged with CO_2_ before finally pressurizing to 15 bar and heating to 1200 °C at a rate of 10 °C·min^–1^. During the heating process, the stirring speed was 200–300 rpm until the temperature was reached in the vessel. Then, the stirring speed was increased to 1000 rpm to start the reaction. The reactor was equipped with a system to extract small quantities of the reaction mixture. The reaction mixture was separated from the catalyst using 0.2 μm nylon filters. ^1^HNMR samples were prepared with 50 μL of the reaction mixture and 600 μL of CDCl_3_, containing 0.03% (v/v) of trimethylsilane (TMS) as an internal standard. The analysis was performed by ^1^H NMR spectroscopy on a 300 MHz Bruker spectrometer.

## Supporting Information Available

The Supporting Information contains additional characterization, XRD, SEM, and ICP (PDF)
