# Supplementary material for: Leaching in Specific Facets of ZIF-67 and ZIF-L Zeolitic Imidazolate Frameworks During the CO2 Cycloaddition with Epichlorohydrin
Source: Chem Mater. 2023 Jan 3;35(2):692–9. doi: 10.1021/acs.chemmater.2c03374 (PMC10373435; doi:10.1021/acs.chemmater.2c03374)
Supplement: Supplementary file 1 — cm2c03374_si_001.pdf [file cm2c03374_si_001.pdf]

Leaching in specific facets of ZIF-67 and ZIF-L Zeolitic Imidazolate Frameworks during the CO_2 cycloaddition with epichlorohydrin

*Jose J. Delgado-Marín[#], Alejandra Rendón-Patiño[‡], Vijay Kumar Velisoju[‡], Gadde Sathish Kumar[‡], Naydu Zambrano[‡], Magnus Rueping[‡], Jorge Gascón[‡], Pedro Castaño[‡], Javier Narciso[#], Enrique V. Ramos-Fernandez[#] **

* Instituto de Materiales and Departamento de Química Inorgánica, Facultad de Ciencias, Universidad de Alicante, Apdo. 99, 03080 Alicante (Spain) email: enrique.ramos@ua.es

[#] Instituto de Materiales and Departamento de Química Inorgánica, Facultad de Ciencias, Universidad de Alicante, Apdo. 99, 03080 Alicante (Spain)

[‡]KAUST Catalysis Center, Advanced Catalytic Materials, King Abdullah University of Science and Technology, Thuwal 23955, (Saudi Arabia)

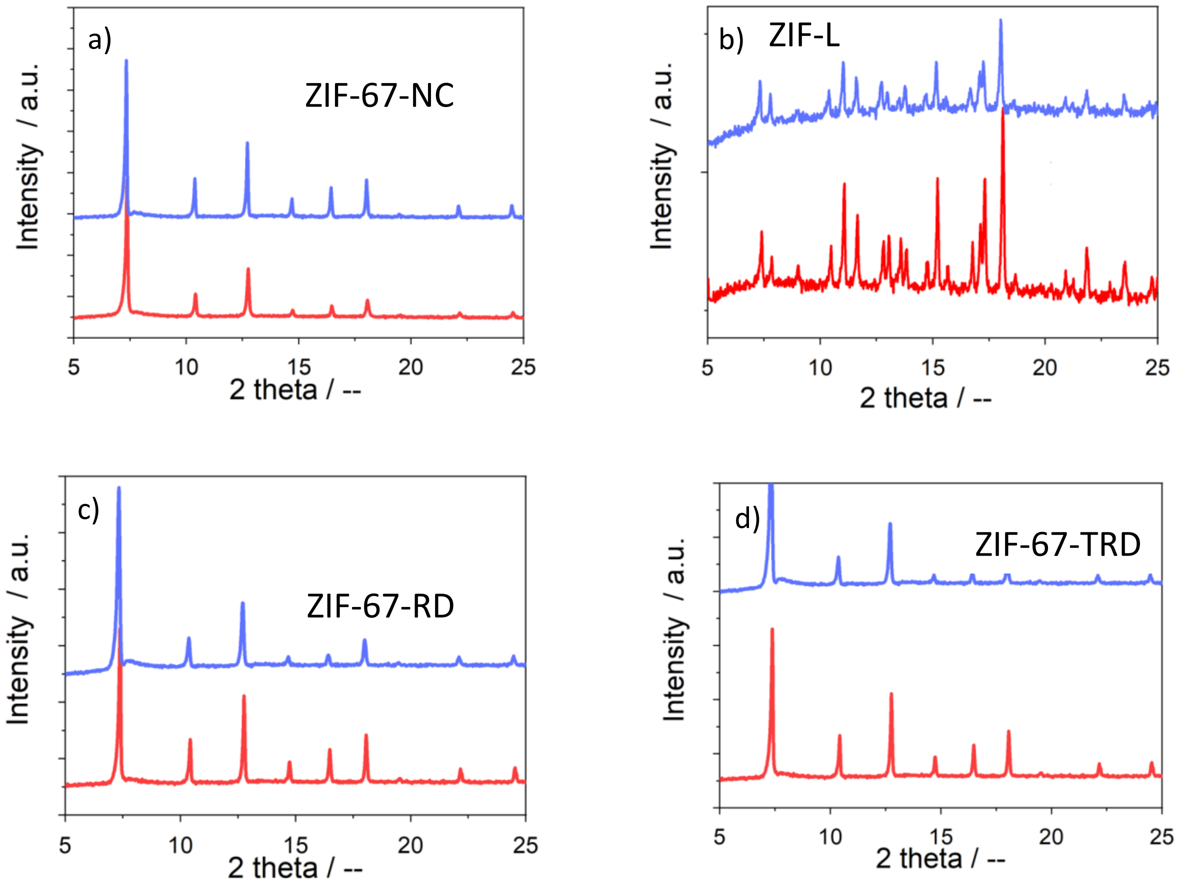

Figure S1. The figure shows the diffraction patterns of a) ZIF-67-NC, b) ZIL-L, c) ZIF-67-RD and d) ZIF-67-TRD. The blue lines belong to the samples used once in reaction; the red lines belong to the fresh samples.

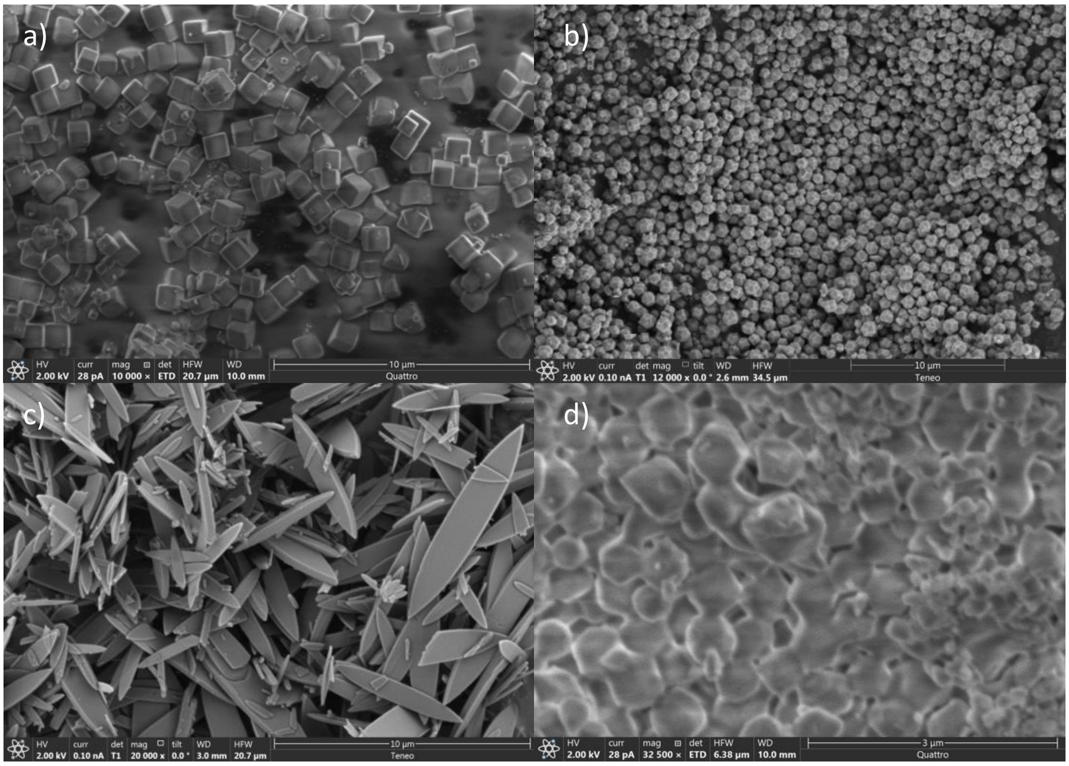

Figure S2. SEM images of a) ZIF-67-NC, b) ZIF-67-RD, c) ZIF-L and d) ZIF-67-TRD.

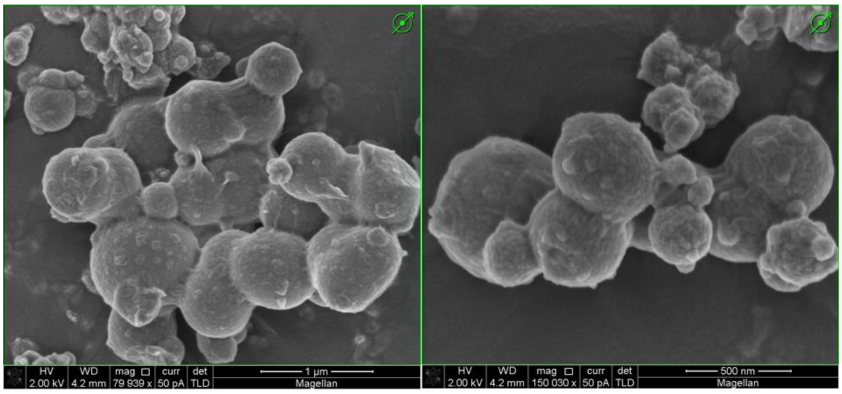

Figure S3. SEM images of the solid collected after the reaction when a mixture of $Co(NO_3)_2$ and 2-methylimidazole are used

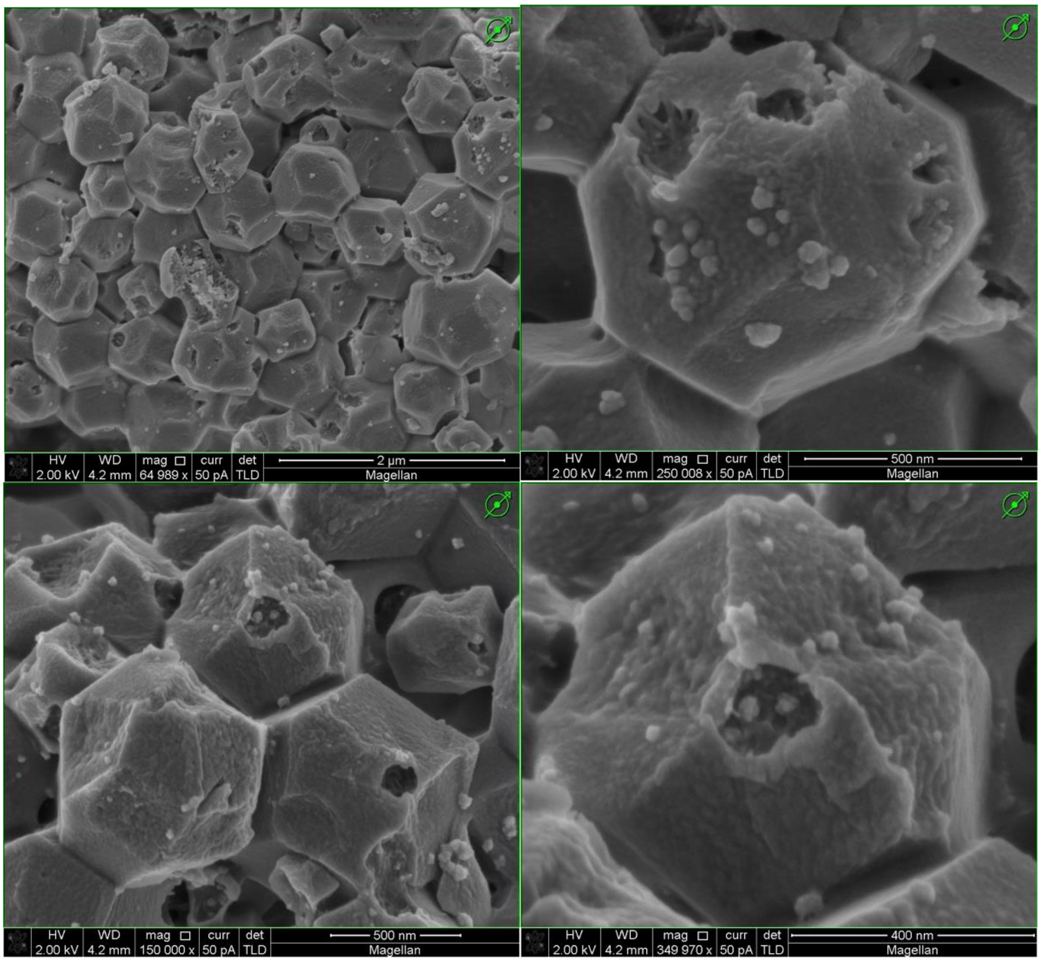

Figure S4. SEM images of the sample ZIF-67-RD after being used.
